# Earthworm genomes, genes and proteins: the (re)discovery of Darwin's worms

**DOI:** 10.1098/rspb.2008.1510

**Published:** 2008-12-16

**Authors:** S.R. Stürzenbaum, J. Andre, P. Kille, A.J. Morgan

**Affiliations:** 1 Pharmaceutical Sciences Division, School of Biomedical & Health Sciences, King's College LondonLondon SE1 9NH, UK; 2 Cardiff School of Biosciences, Cardiff UniversityPark Place, Cardiff CF10 3US, UK

**Keywords:** Darwin, earthworms, evolution, genotypes, biogeography, transcriptomics

## Abstract

Small incremental biological change, winnowed by natural selection over geological time scales to produce large consequences, was Darwin's singular insight that revolutionized the life sciences. His publications after 1859, including the ‘earthworm book’, were all written to amplify and support the evolutionary theory presented in the *Origin*. Darwin was unable to provide a physical basis for the inheritance of favoured traits because of the absence of genetic knowledge that much later led to the ‘modern synthesis’. Mistaken though he was in advocating systemic ‘gemmules’ as agents of inheritance, Darwin was perceptive in seeking to underpin his core vision with concrete factors that both determine the nature of a trait in one generation and convey it to subsequent generations. This brief review evaluates the molecular genetic literature on earthworms published during the last decade, and casts light on the specific aspects of earthworm evolutionary biology that more or less engaged Darwin: (i) biogeography, (ii) species diversity, (iii) local adaptations and (iv) sensitivity. We predict that the current understanding will deepen with the announcement of a draft earthworm genome in Darwin's bicentenary year, 2009. Subsequently, the earthworm may be elevated from the status of a soil sentinel to that elusive entity, an ecologically relevant genetic model organism.

## 1. Introduction

The subject may appear an insignificant one, but we shall see that it possesses some interest; and the maxim ‘*de minimis lex non curat*’ (the law is not concerned with trifles) does not apply to science. (Charles Darwin 1881, page 2)

**Figure 1:**
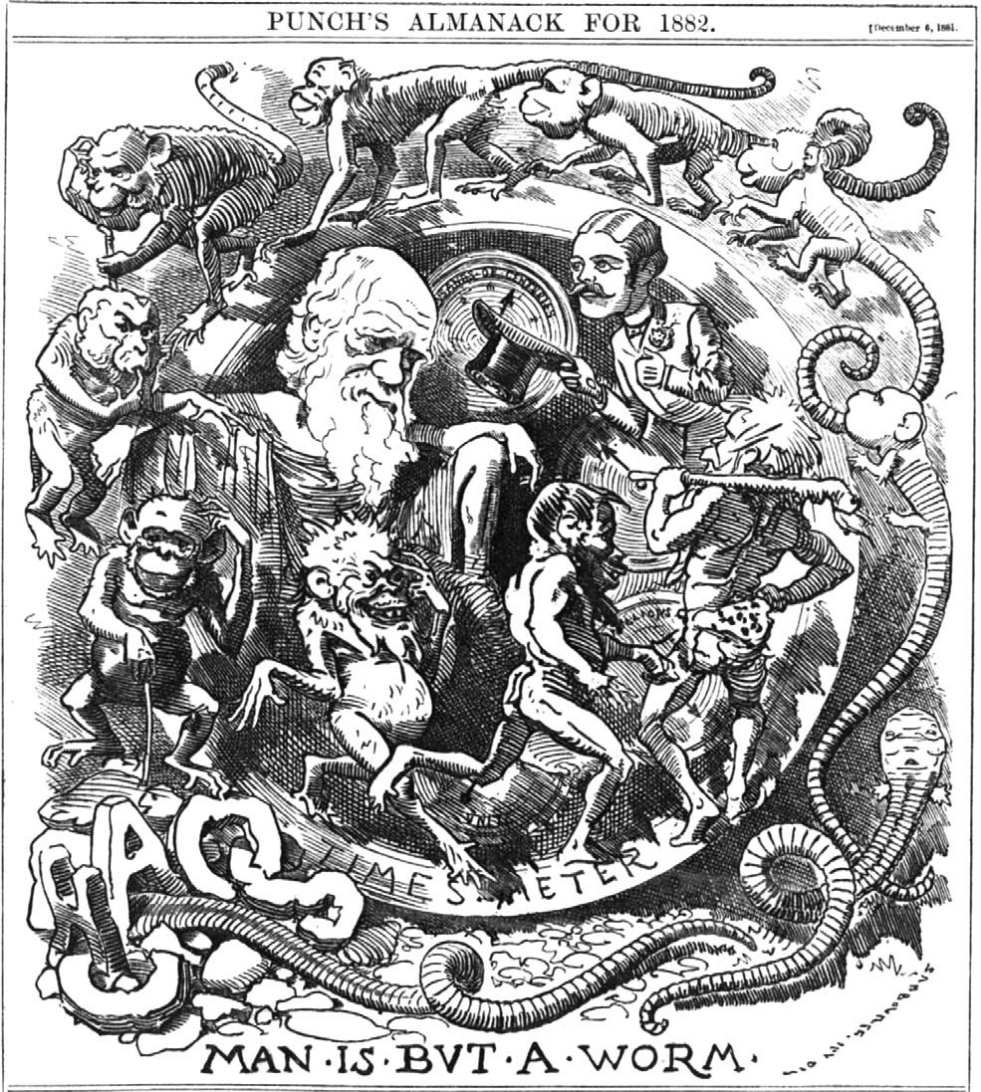
*Punch*'s almanac for 1882: ‘Man is but a worm’, published in *Punch Magazine* on 6 December 1881. The satirical cartoon shows how Darwin has evolved from chaos, over earthworms to respectable gentleman.

There is a wonderful symmetry to Charles Darwin's (1809–1882) formal interest in earthworms. His first paper described the activities of earthworms and was the central theme of his presentation to the Geological Society in 1837 (Darwin 1838), 22 years before *On the* *Origin of species by means of natural selection* (Darwin 1859), and his last book, a monograph on the formation of mould through the action of worms (Darwin 1881, page 3), was published 22 years after the *Origin*. Despite Darwin's ‘fear that the subject of it will not attract the public’, the earthworm book proved to be the most successful publication during his own lifetime, with 3500 copies sold within a matter of days (Browne 2002). An iconic cartoon published in *Punch* on 6 December 1881 depicts Darwin surrounded by a series of images of an earthworm emerging from the primordial soup and evolving from ape to a human embodied by himself (figure 1). The illustration is a humorous construct, but an examination of the earthworm structure and function reveals cells and tissues and cell types with vertebrate counterparts. Earthworms (phylum: Annelida; class: Clitellata; subclass: Oligochaeta; order: Opisthopora) are coelomate protostomes, possessing an anatomically and functionally differentiated alimentary canal with brush-bordered absorptive epithelia, a closed blood circulation with haemoglobin in free suspension, an organized nervous system with cephalic ganglia and neurosecretory activities, a multifunctional tissue (the chloragog) for which carbohydrate metabolism and storage properties are reminiscent of mammalian hepatocytes, a series of paired tubules (nephridia) in each segment with renal urine-forming functions, and a systemic immune system comprising leucocyte-like cells (coelomocytes).

**Figure 2:**
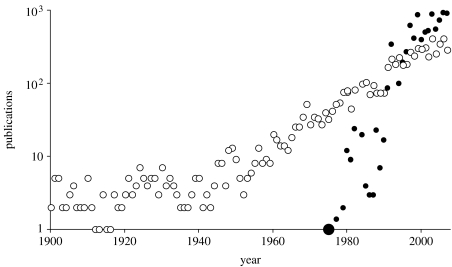
Number of papers published annually between 1900 and 2008 as listed in ISI Web of Knowledge (http://wok.mimas.ac.uk). While publications on earthworms (white circles) have accumulated throughout the century, the first paper on *C. elegans* was published in 1974 (enlarged black circle). Note that since the release of the *C. elegans* genome in 1998, numbers of *C. elegans* papers (black circles) have overtaken papers published on earthworms.

Contemporary biologists have enthusiastically embraced Darwin's concept of the exploitation of specific ‘model’ organisms to explore fundamental aspects of life processes. Intriguingly, the organism in the vanguard of the genomics era was not the earthworm, but the nematode roundworm *Caenorhabditis elegans*. The development of a comprehensive suite of tools to unravel the basis of biology, biochemistry and genetics has generated an exponential rise in published output over the last 10–20 years (figure 2). However, based on the citation reports listed in ISI Web of Knowledge, *C. elegans* research is strong in subject areas such as biochemistry and cell, molecular and developmental biology, but rather underdeveloped in areas that encompass ecology, soil and environmental sciences (table 1). But Darwin's earthworms have by no means been neglected by the scientific community during the 125 years or so since the publication of his milestone monograph (figure 2). Indeed, earthworms have been studied to address key issues in environmental and ecosystem sciences, an effort that continues relentlessly (table 1).

Until recently, the body of earthworm genetics research was negligible owing to the lack of a detailed molecular genetic knowledge base. However, the trend is changing. During the last decade, we have seen the development of a wide spectrum of molecular genetic resources for earthworms ranging from evolutionary tools, such as mitochondrial DNA markers (COI and COII; Heethoff *et al.* 2004; Chang *et al.* in press; King *et al.* 2008), amplified fragment length polymorphism (AFLP) profiling (King *et al.* 2008) and a suite of microsatellite markers (Harper *et al.* 2006; Velavan *et al.* 2007), and functional genomic tools comprising more than 20 000 expressed sequence tag (EST) sequences, microarray-based transcriptomic profiling (Sturzenbaum *et al.* 2003; Lee *et al.* 2005; Gong *et al.* 2008; Owen *et al.* 2008) and NMR-based metabolic fingerprinting (Bundy *et al.* 2008; Jones *et al.* 2008). In this short review, we propose to adopt the hypothetical (perhaps presumptuous) position of marshalling in a selective manner some of the newly discovered genetic information in the spirit of continuing Darwin's quest to use features of earthworm biology to further our understanding of global evolutionary processes.

## 2. Biogeography: flightless and legless peregrination

They inhabit the most isolated islands; they abound in Iceland, and are known to exist in the West Indies, St. Helena, Madagascar, New Caledonia and Tahiti. In the Antarctic regions, worms from Kerguelen Land have been described … and I found them in the Falkland Islands. (Charles Darwin 1881, pages 120–121)

Differences in the geographical distribution pattern of taxa (table 2), their presence at one location but absence from another, delineates differences both in the evolutionary history of organic life and in planetary history. The absence of a taxon may not reflect its innate (in)capacity to exploit local resources but the more prosaic matter of an inability to reach the location (James 2004). Thus, it has been postulated that the biogeography of modern taxa, specifically earthworms, should reflect the history of macro events—such as continental drift, island formation, global climate changes—and so would resonate with Darwin's core statements. Of course, aspects of anthropochory should not be disregarded as an important factor in the dispersal route of (peregrine) species of earthworms. Indeed, molecular genetic tools, exploiting allozymic, nuclear and mitochondrial markers, are increasingly being exploited to improve the present uncertainties and controversies in the biogeography of the terrestrial Oligochaeta (Jamieson *et al.* 2002; James 2004), as well as to describe the routes of dispersal of species across the Baltic Sea and onto North European islands (Terhivuo & Saura 2006). Moreover, observations on the genetics of earthworm populations that have evolved cold-tolerance traits in response to seasonal sub-zero temperatures in northern latitudes (Hansen *et al.* 2006; Holmstrup *et al.* 2007) are contributing to an understanding of how soil-dwelling members of the taxon are able to colonize such inhospitable habitats.

## 3. Earthworm diversity: unfathomed depths

Earth-worms are distributed throughout the world under the form of a few genera, which externally are closely similar to one another. (Charles Darwin 1881, page 8)

When and where soil moisture, nutrient status and temperature are favourable, a habitat can support a few, but often no more than six, species of earthworm (Hendrix & Bohlen 2002). This implies that the functional redundancy may be commonplace within large decomposer assemblages, such as the earthworm (Setälä *et al.* 2005). However, because species belonging to different ecological groups (namely the surface-dwelling epigeics, the soil- and plant root-feeding endogeics or the underground burrowing and feeding anecic earthworms) have different effects on the soil processes, the concept of functional redundancy in earthworms must, by implication, refer not indiscriminately to local species richness but to whether representation of the broad ecological groupings is maintained. Studies have shown that the earthworm communities are less species rich, with a predominance of endogeic species, in agroecosystems in Mexico, Peru and India (Fragoso *et al.* 1997). Casual observations indicate that the epigeic species with their trophic dependence on a litter layer are not widespread in agricultural systems, and are often the sole ecological group present on the typically shallow soils associated with abandoned mining and industrial sites. Therefore, the site-specific relationship between the genetic and local diversity of an earthworm community warrants serious consideration. A recent study (Lentzsch & Golldack 2006) reported that the intraspecific genetic variability in the endogeic *Aporrectodea caliginosa* was not related to the soil composition or the physical features of the landscape but was strongly influenced by the earthworm species richness. This study raises the possibility that the highly heterogeneous nature of undisturbed soils, coupled with the relatively low dispersal rates of earthworms, may combine to promote sympatric speciation.

The absence of direct competitors may allow a species to expand its functional or Hamiltonian niche (*sensu* Setälä *et al.* 2005), perhaps towards those of species belonging to missing or underrepresented ecophysiological groups, and lead to the establishment of local intraspecific genetic heterogeneities. Johansson (2008) has modelled the interactions between an organism's ecology and its evolutionary responses to evolutionary change, and has concluded that the interspecific competition within a resource landscape can reduce rates of local adaptation. It would, therefore, be very instructive to examine the genetic constitutions of exotic invasive earthworm populations. In each case, where exotics have become firmly established, and effectively exclude the natives, there is evidence of habitat disturbance, leading to complete or partial elimination of the resident community, followed by the chance of (perhaps successional) introduction of one or more exotic species (Hendrix & Bohlen 2002; Hale *et al.* 2005).

**Figure 3:**
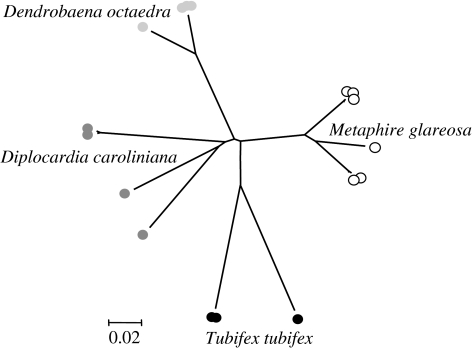
Phylogenetic analysis of species diversity within a range of Annelida. Representative COI sequences were selected from GenBank to illustrate maximal diversity with four annelid species: *T. tubifex* (EF179544.1, EF179543.1 and AF534866.1); *Metaphire glareosa* (AY960803.1, AY962167.1, AY962168.1, AY962169.1, AY962178.1 and AY962179.1); *Dendrobaena octaedra* (EU035478.1, EU035481.1, EU035484.1, EU035487.1, EU035488.1, EU035492.1 and DQ092895.1); and *Diplocardia caroliniana* (EF156651.1, EF156658.1, EF156659.1 and EF156661.1). The tree was constructed using the distance-based neighbour-joining algorithm, based upon *p* distance.

Classical taxonomy is based on the examination and comparisons of morphological structures. The body plan of oligochaete worms, largely devoid of prominent external appendages other than the secondary sexual structures decorating the evolutionary innovation of metameric segmentation, limits the scope of morphological taxonomy. Application of enzyme electrophoresis in the 1980s and 1990s increased the information in many topics of earthworm research, such as in taxonomy (Øien & Stenersen 1984), allozyme diversity in amphigonic and polyploid strains (e.g. Cobolli Sbordoni *et al.* 1987), diversity and regional adaption of clone pools in parthenogenetic species (Terhivuo & Saura 1990, 1993) and temporal variability of clones in parthenogens (Jaenike & Selander 1985). The advent of molecular genotyping tools for earthworms (Chang *et al.* in press) has begun to reveal hitherto unsuspected degrees of ‘intraspecific’ genetic diversity that represent potential cases of cryptic speciation, defined as morphologically similar but genetically distinct sibling species (Rocha-Olivares *et al.* 2004). An analysis of mitochondrial cytochrome oxidase subunit I (COI) sequences of the small number of contrasting oligochaete ‘species’ deposited in genetic databases (National Center for Biotechnology Information, GenBank, DNA Data Bank of Japan) demonstrates multiple genetically differentiated lineages within each species cluster (figure 3). It is possible that the Oligochaeta are particularly prone to sympatric speciation. For example, Sturmbauer *et al.* (1999) identified that the mitochondrial 16S rDNA of the freshwater worm *Tubifex tubifex* could be differentiated into five major lineages (separated by genetic distances of up to 13%), providing strong evidence for the presence of cryptic speciation. Likewise, COI genotyping on the representatives of the British earthworm fauna indicate that at least four of the eight species contain two to three distinct lineages that may diverge by over 12 per cent (King *et al.* 2008). Another notion supporting the fact that earthworms are genetically heterogeneous is that both the amphigonic and polyploid strains can exist within a species as shown by surveys on chromosomal status of populations (Casellato 1987). The origin of this diversity is not known, but the convergent postglacial invasion of multiple genotypes from geographically isolated refugia of southern Europe has been offered as a plausible explanation (King *et al.* 2008). Whatever factors gave rise to the genetic diversity of earthworms, the ecological and evolutionary implications of its existence are wide-ranging. In short, Darwin was correct in saying that earthworms are closely similar to each other, but he would have been stunned at how modern molecular techniques are able to distinguish between the many different species (belonging to a large number of different genera) and provide a compelling case for including leeches and branchiobdellids within the Oligochaeta (Jamieson *et al.* 2002).

## 4. Evolutionary conservation: if it ain't broke…

Pancreatic juice emulsifies fat, and we have just seen how greedily worms devour fat; it dissolves fibrin, and worms eat raw meat; it converts starch into grape-sugar with wonderful rapidity, and … the digestive fluid of worms acts on starch. (Charles Darwin 1881, page 37)

Evolutionary conservation is echoed at the genetic level. Of the 8129 unique ESTs previously isolated from the earthworm *Lumbricus rubellus* (Sturzenbaum *et al.* 2003; Owen *et al.* 2008), a cohort of 1728 gene objects (i.e. over 21%) display significant homologies to counterparts identified in the genomes of the fruitfly (*Drosophila melanogaster*), the nematode (*C. elegans*) and humans (*Homo sapiens*). This underlines the notion that key biological and metabolic pathways are conserved within the majority of eukaryotic organisms. Perhaps more interesting are the cohorts that display homology only between the earthworm and the fruitfly (68 genes), earthworm and nematode (49 genes), or earthworm and humans (220 genes). That more earthworm genes are conserved between earthworms and humans provides anecdotal support of the original *Punch* cartoon strap line: ‘man is but a worm’. Even before the dawn of the genetics era, let alone the genomics era, Darwin presciently anticipated the surprising revelation of high degrees of evolutionary conservation within the animal kingdom.

The recent availability of substantive genetic datasets has been essential for the execution of far-reaching phylogenetic analyses and the attempt to answer questions relating to fundamental evolutionary relationships between the various animal phyla (Philippe *et al.* 2005). This fresh approach has challenged some evolutionary classifications, dogmas based on developmental and anatomical features described some 150 yr ago (Jones & Blaxter 2005). However, questions remain unresolved at least until equity of genetic knowledge across the full diversity of eukaryotes is achieved.

Even a casual review of significant homologies derived for earthworm cDNAs raises a number of intriguing evolutionary questions. For example, the presence of chitin-like proteins outside the phylum Arthropoda may initially seem perverse, until the reader is reminded that the soft-bodied earthworm possesses chitinized chaetae/setae, gizzard and egg capsules (Sims & Gerard 1999). Likewise, the identification of bone morphogenic protein (LRC00553) in earthworms requires an explanation. Darwin described at some length the presence of a mineralizing organ, namely the calcium carbonate-excreting calciferous gland (Gago-Duport *et al.* 2008; Lee *et al.* 2008), in certain unspecified lumbricid earthworm species. These two examples illustrate how evolution can retain and adapt key pathways for innovative purposes, but our understanding of how comparative genomics impinges functionally on comparative physiology is presently limited. The genome of the earthworm *L. rubellus*, for example, encodes a rhodopsin kinase (LRC00925). This enzyme is a homologue of the ‘eye-specific’ photoreceptor in flies (Doza *et al.* 2005), other invertebrates and vertebrates. Moreover, the earthworm genome contains a recoverin homologue (LRC00100), a Ca^2+^-binding protein that participates in light adaptation by imposing an inhibitory constraint on rhodopsin kinase (Kawamura *et al.* 1993). The finding that essential components of the molecular machinery of photoreception is present in a negatively phototropic metazoan organism without recognizable eyes would almost certainly stimulate in Darwin an interest in the field of evolutionary developmental biology (evo-devo; Carroll 2006).

## 5. Ecotoxicology: chemical warfare and molecular diplomacy

They are easily killed by salt-water … acetic acid is so deadly a poison to worms that … a glass rod dipped into this acid and then into a considerable body of water in which worms were immersed killed them quickly. (Charles Darwin 1881, pages 121,159)

The keystone role played by earthworms within terrestrial ecosystems, established in part by Darwin's own research, necessitates a detailed understanding of how environmental change, either anthropogenic or geogenic, impacts on survival and fecundity. This premise has been the foundation for the exploitation of earthworms as ecotoxicological sentinel organisms for the soils. Currently, the Organisation for Economic Co-operation and Development (OECD) has a number of testing regimes by which chemical-induced lethal and sublethal earthworm toxicosis can be used to aid informed risk assessments for environmental release. To date, the ECOTOX data resource (USEPA 2007) records 10 000 separate toxicological studies that employ earthworms as test organisms. This canon of literature provides an invaluable resource for comparative toxicology; however, the majority of the archived studies use mortality as an endpoint rather than subtler endpoints such as reproductive output or complex life-history parameters, which are prerequisites for sound demographic modelling.

**Figure 4:**
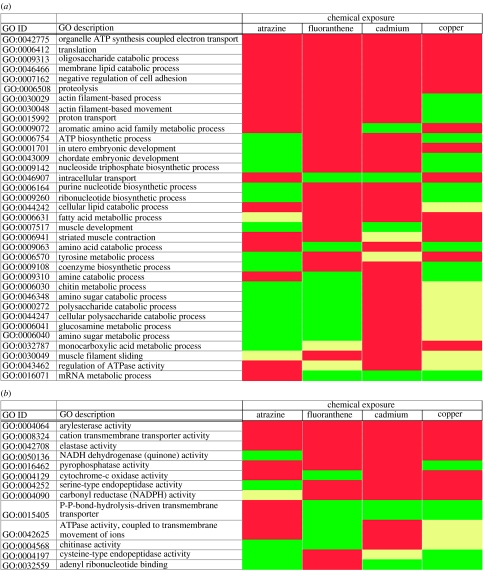
Comparative analysis of the representation of functional categories within transcriptomes challenged with organic and inorganic pollutants. Transcripts for which expression is statistically altered by exposure to the pesticide atrazine, the polycyclic hydrocarbon (PAH) fluoranthene and inorganics cadmium and copper (Bundy *et al.* 2008; Owen *et al.* 2008) were analysed for the over-representation of ontological terms associated with (*a*) ‘biological process’ and (*b*) ‘molecular function’. A list of human homologues were used to annotate the earthworm genes by homology using DAVID (Dennis *et al.* 2003), and the probability of the resultant occurrence of ontological terms at level 5 calculated using the EASE algorithm (Hosack *et al.* 2003). Ontological categories are displayed where over-representation occurs (*p*<0.1) and the number of genes present in the category is more than 1 (to avoid stochastic observations), observed in response to one of the chemical challenges. Exposures where gene representation in an ontology category shows less than two genes are shown in yellow, while those displaying two or more genes but for which significance is greater than 0.1 are shown in green, and those where two or more genes are present and have a significance less than 0.1 are displayed in red.

The exploitation of genomic tools in soil ecotoxicology, with earthworms at the forefront of the enterprise because of their ecological status, promises to provide mechanistic insights into the modes of action underpinning the toxicosis of specific chemical residues. In addition, the tools will provide diagnostic signatures associated with the disruption of key biological processes, such as reproduction and growth. A number of recent studies have employed both transcriptomics (Gong *et al.* 2007, 2008; Owen *et al.* 2008) and metabolomics (Bundy *et al.* 2008) to investigate the physiological shifts that occur in response to organic and inorganic pollutants. An incisive review of this material reveals that there is a substantial overlap between the responses to different chemicals (van Straalen & Roelofs 2008) and, tellingly, remarkable degrees of interaction (overlap, synergism and antagonism) in the transcriptomic profiles induced by a range of chemical and physical stressors (Roelofs *et al.* 2008). From an ecotoxicogenomics perspective, the datasets also proclaim the opportunities, perhaps through combining observations on ‘global’ transcriptome profiles with the phenotypic description provided by metabolomics, to select compound-specific responses for rapid and environmentally meaningful assessments of chemical exposures with in-built predictive capabilities (van Straalen & Roelofs 2008). The enrichment of specific ontological categories associated with challenges to four contrasting toxic chemicals (figure 4) lends credence to this proposition, but the interpretation of such findings is limited because of our present incomplete knowledge of earthworm molecular genetics and the absence of direct functional annotations for earthworm genes.

Ultimately, molecular genetic approaches for environmental monitoring will prove to be faster, more sensitive, more stressor-specific, more predictive, more cost-effective and/or more informative than the standard earthworm tests in common usage today. The scientific community and regulatory agencies eagerly anticipate the breakthrough that will transform classical ecotoxicology into true ecotoxicogenomics (Snape *et al.* 2004; Ankley *et al.* 2006).

## 6. Prospects: genetic furrows and nature's plough

The plough is one of the most ancient and most valuable of man's inventions; but long before he existed the land was in fact regularly ploughed, and still continues to be thus ploughed by Earth-worms. (Charles Darwin 1881, page 313)

Prior to Darwin's book, earthworms were considered to be pest animals of the soil. His scientific work was an important milestone in changing this attitude. Darwin gleaned his knowledge of earthworms by a combination of personal observation (including ‘laboratory’ and field experimentation), the assimilation of the works of contemporary European earthworm authorities (such as Eisen, Hoffmeister and Morren), and active correspondence with collaborators in Britain and in the farthest reaches of the Empire. Apart from their convenient accessibility for a man frequently incapacitated by ill health, how do we account for Darwin's particular and long-lasting fondness for earthworms? Stephen Jay Gould (Gould 1982) posits, convincingly in our view, that Darwin's last book has two threads: an explicit description of earthworms and their activities, and an implicit temporal theme emphasizing how studying present events shed light on the historical past, i.e. on evolution.

As mentioned earlier, the exploitation of modern molecular genetic tools is now a routine approach applied to genotype earthworm populations to answer questions relevant to evolution, including the discovery of cryptic species, *per se*, or the identification of genotypic differences that modulate differential phenotypic responses to environmental change (Sturmbauer *et al.* 1999). There is evidence that at least some oligochaetes, such as *Aporrectodea caliginosa trapezoides*, possess high levels of methylated DNA (Regev *et al.* 1998). It is conceivable that earthworms use methylation as a primary mechanism of epigenetic control to promote phenotypic variation and plasticity, which are requisites for the colonization of geochemically diverse soils (e.g. in terms of pH, trace metal concentrations). Getz (2006) succinctly summarized the challenges heralded by these new insights: ‘…we continue to put too much store in a gene-centric view of the evolutionary process. Furthermore, the modern synthesis … does not account for … the appearance of Lamarck's ghost in the influence of the environment on DNA methylation and gene expression’. Mapping the epigenome (Baylin & Schuebel 2007), particularly in keystone environmental engineers and sentinels, such as the earthworm, will soon define the contribution of epigenetic variation to the evolution of ecologically relevant phenotypic traits in response to environmental stress (Szyf 2007) and in establishing how invasive species are successful (Pérez *et al.* 2006).

However, the largest leap is impending. With the onset of a recently funded sequencing approach using high end 454 and Solexa massively parallel sequencing platforms and targeted gap-filling by bacterial artificial chromosome sequencing, the earthworm-studying community is awaiting the release of the draft earthworm genome sequence by the end of 2009. The announcement of the genome sequence is likely to appeal to classical biologists/zoologists, technologists, bioinformaticians, molecular biologists/geneticists, evolutionary biologists, ecotoxicologists, legislators and biomarker scientists. It is hoped that this will result in an explosive growth of research output, not unlike that experienced in *C. elegans* research immediately after its genome became available. In any case, Darwin's earthworm will be propelled from being a sentinel soil organism to being a genetic model organism for environmental soil science.

## Figures and Tables

**Table 1:** *Caenorhabditis elegans* and earthworm papers published between 1900 and August 2008 listed in ISI Web of Knowledge (http://wok.mimas.ac.uk). (Note that when separated into individual categories it is apparent that *C. elegans* research is dominated by research into biochemistry and cell, molecular and developmental biology, and earthworm research is geared towards soil science, ecology and environmental science, a trend that is also supported by the citation index (h-score).)

		ISI Web of Knowledge categories: biochemistry and cell, molecular and developmental biology			ISI Web of Knowledge categories: soil science, ecology and environmental science		
			
	total	papers	average citation per item	h-score	papers	average citation per item	h-score
earthworms	6437	723	14.6	45	4216	13.0	69
*C. elegans*	9142	7243	40.9	204	43	10.3	12

**Table 2:** The regional distributions of the 10 recognized major families of terrestrial earthworms (phylum: Annelida; class: Clitellata; subclass: Oligochaeta; order: Opisthophora). (Redrawn from the secondary source—Hendrix & Bohlen 2002.)

family	geographical region of origin
Ailoscolecidae	Europe
Eudrilidae	Africa
Glossoscolecidae	Central America, South America
Hormogastridae	Mediterranean
Komarekionidae	North America
Kynotidae	Madagascar
Lumbricidae	Europe, North America
Megascolecidae	Africa, Central America, North America, South America, Asia, Madagascar, Oceania
Microchaetidae	Africa
Ocnerodrilidae	Africa, Central America, South America, Asia, Madagascar
